# Empowering the Patient With Chronic Obstructive Pulmonary Disease: Optimizing Inhalation Technique

**DOI:** 10.7759/cureus.77019

**Published:** 2025-01-06

**Authors:** Sofia Castro, Jorge Moura, Filipa Dias, Diogo Magalhães, Inês Vasconcelos, António Abreu, Mariana Rio, Inês Sousa, Maria Corral, Edmundo Ferreira

**Affiliations:** 1 Porto Centro Family Health Unit, Local Health Unit São João, Porto, PRT

**Keywords:** chronic obstructive pulmonary disease, dry powder inhalers, empowerment, inhalation technique, metered dose inhalers

## Abstract

Introduction

Chronic obstructive pulmonary disease (COPD) is a heterogeneous lung disease characterized by chronic respiratory symptoms. Validated tools such as the modified Medical Research Council (mMRC) and COPD Assessment Test (CAT) questionnaires are commonly used to assess these symptoms. In Portugal, it is estimated that COPD affects approximately 14.2% of individuals aged 40 and over. Compliance with inhalation therapy remains a significant challenge. Inconsistent and incorrect use of inhalers contributes to suboptimal disease control and deteriorates the patients’ quality of life. Regular evaluations provide a valuable opportunity to monitor symptom progression, assess therapeutic compliance, and understand patients' beliefs and expectations about their treatment.

Objective

The primary aim of this study was to enhance the correct use of inhalers at the Porto Centro Family Health Unit by at least 20% of the COPD patients included. The secondary goal was to evaluate how inhaler therapy compliance and proper technique impact the symptomatic control of COPD, as measured by the mMRC and CAT scales.

Methods

A prospective observational cohort study was developed, with an intervention component. Data were gathered through random sampling of patients diagnosed with COPD. Participants were invited for an initial evaluation, where they were educated about the disease, instructed on the correct inhalation technique, and assessed for both symptom severity and inhaler technique. They were then re-assessed at a follow-up visit. Descriptive and analytical statistical methods were applied to compare and interpret the results. This study was approved by the Ethics Committee of Local Health Unit São João.

Results

A total of 60 patients were randomly selected for participation. After applying exclusion criteria, refusal to participate, and missed appointments, 19 participants completed the study. Approximately two-thirds of the patients initially failed at least one step of the inhalation technique. Following the intervention, 79% of the patients were able to correctly perform all steps, reflecting a 47% improvement. Symptom assessment using the CAT questionnaire showed scores ranging from 8 to 21 (median = 8) at the first visit, which improved to a range of 0 to 21 (median = 4) at the follow-up visit. In terms of mMRC scores, only one patient showed a worsening of dyspnea classification, three patients improved, and the remaining patients had stable classifications.

Discussion

Inhalation therapy is crucial for effective COPD management. Following the intervention, a 47% improvement in inhaler technique was observed, which could lead to better disease control, fewer hospital admissions, and an enhanced quality of life. Although there are limitations such as the small sample size, the study demonstrated symptom improvement between the first and second visits, potentially indicating improved disease management.

Conclusions

This study highlights the impact that a focused intervention in managing COPD can have on improving patient outcomes and quality of life.

## Introduction

Chronic obstructive pulmonary disease (COPD) is a heterogeneous lung disease characterized by chronic respiratory symptoms due to airway and/or alveolar abnormalities that cause persistent, and often progressive, airflow obstruction. Symptom assessment can be done using validated questionnaires such as the modified Medical Research Council dyspnea scale (mMRC) and the COPD Assessment Test (CAT) [1-4].

In Portugal, it is estimated that COPD affects approximately 14.2% of individuals over 40 years [5]. It is a major cause of morbidity and mortality and is one of the three leading causes of death worldwide [6]. It is associated with high economic costs [7], particularly in relation to poor disease control and consequent hospitalizations. Inhaled therapy is the one that provides the best results in the management of patients with COPD.

Compliance refers to the extent to which a patient’s behavior matches the prescriber’s recommendations [8], and it is a challenge in any chronic disease, including inhalation therapy in COPD [9]. Research has shown high non-compliance rates, above 50%, ranging from 22% to 93% in some studies. On average, more than two-thirds of patients miss/fail at least one step while using their inhaler [1].

Incorrect and/or inconsistent use of inhalers leads to poor disease control, with worsening lung function, increased symptoms, higher frequency and severity of exacerbations, often requiring hospitalization, and an overall worsening quality of life [10].

Periodic primary care evaluations are an important opportunity to assess disease progression, therapeutic compliance, and patient expectations and beliefs regarding treatment, thus preventing disease development [11-12]. Explaining the disease and the impact of treatment compliance and correct inhalation technique may lead to optimal disease management, with a significant improvement in quality of life [10].

The main objective of this study was to improve the inhalation technique in patients with COPD at the Porto Centro Family Health Unit, by at least 20% of the patients included. The secondary objective was to evaluate its impact on the symptomatic control of these patients using the mMRC and CAT questionnaires.

## Materials and methods

A prospective observational cohort study was conducted as an intervention project. Data were obtained through random sampling of patients registered at Porto Centro Family Health Unit with a diagnosis of COPD.

**Figure 1:**
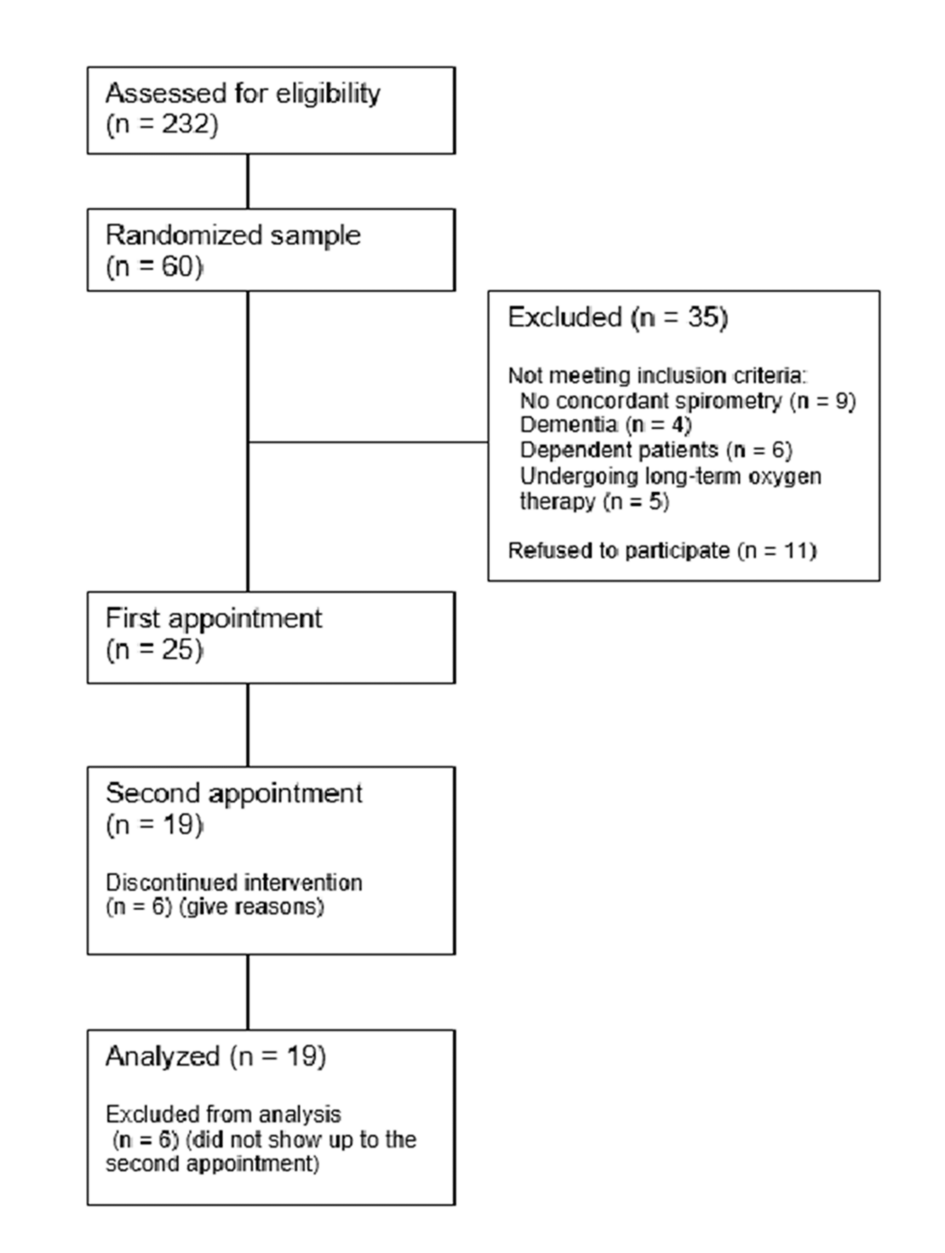
Extraction of the COPD patient sample after applying inclusion and exclusion criteria. COPD: Chronic obstructive pulmonary disease.

Patients with an active diagnosis of COPD, coded as 'R95' using the International Classification of Primary Care 2 (ICPC-2), were identified by consulting a Portuguese medical database called 'Módulo de Informação e Monitorização das Unidades Funcionais' (MIM@UF), with a total of 232 patients in this Health Unit. A randomized sample was subsequently obtained, with the selection of 60 patients. To randomize, a computer program generated a randomized sequence of integers and then extracted the patients in Excel that had that number attributed. The diagnosis of COPD was confirmed through verification of medical records, such as the existence of concordant spirometry. Those who did not meet these criteria were excluded from the study. Patients who had dependency, were undergoing long-term oxygen therapy, and/or had dementia were excluded (Figure 1).

Patients were contacted by telephone by their family doctors, using a standard script for all, inviting them to an appointment and asking them to bring their inhaler and, if applicable, the spacer. At each appointment, doctors used the same written guide to homogenize the care provided. The intervention consisted of demonstrating the correct inhalation technique step-by-step and explaining the importance of consistent and correct treatment.

At the first appointment, patients were observed by one of the research team members at Porto Centro Family Health Unit.

Doctors involved explained COPD pathology (in adapted terms) and the importance of the correct use of inhalers. Two validated questionnaires were given to the patients to answer - mMRC and CAT (Appendix 1). Adherence to therapy was assessed, and the correct inhalation technique was reviewed according to the type of inhaler used, using a checklist [13] to assess their knowledge and level of compliance. It was demonstrated to each patient how to use their inhaler correctly. The second appointment was scheduled, preferably four to six weeks later.

At the second appointment, the mMRC and CAT questionnaires were handed out again, and knowledge regarding the inhalation technique was assessed using the same checklist as in the first appointment. In both appointments, patients who were active smokers were encouraged to quit smoking, however, it was not performed as a targeted approach, since it was not the aim of the study. Data were collected and recorded on Microsoft Excel.

The protocol was designed at the beginning of February 2024 and approved by the Ethics Committee Local Health Unit São João (number 111/2024), on July 11, 2024. Data were collected from July 17 until September 30, 2024. The study adhered to all the recommendations of the Declaration of Helsinki (2013).

The impact of our intervention on the number of errors in the inhalation technique and CAT questionnaire scores was assessed using a two-sided Wilcoxon signed-rank test, with a significance level set at 5%. To perform this test, results that showed no change after the intervention were excluded from the analysis.

## Results

Sixty individuals were randomly selected for the study. After applying exclusion criteria, refusal to participate, or absence from appointments, a total of 19 participants were obtained (Table 1).

**Table 1:** Study sample characteristics.

Sample characteristics		
Total of participants (n)	19	
Age	51-83 years (mean = 67)	
Gender	Male 58% (n=11)	
	Female 42% (n=8)	
Smoking history	Nonsmokers 5% (n=1)	
	Smokers 95% (n=18)	Active smokers 47% (n=9)
		Ex-smokers 53% (n=9)
Comorbidities	Asthma 16% (n=3)	
	Obstructive Sleep Apnea Syndrome 11% (n=2)	
	Heart Failure 11% (n=2)	
	Pulmonary Emphysema 5% (n=1)	

The individuals were aged between 51 and 83 years, with an average age of 67 years. The proportion of male participants was 58% (n=11), and female participants 42% (n=8).

The majority, 95% (n=18), had a history of tobacco smoking, with 47% (n=9) being active smokers. The quantity of cigarette smoking ranged from 0 to 80 pack-years (PY), with an average of 43.6 pack-years. Patients with COPD may have other diseases presenting with respiratory symptoms, which can contribute negatively to the control of COPD. In our study, 42% (n=8) had comorbidities associated with COPD: asthma (n=3), obstructive sleep apnea syndrome (n=2), heart failure (n=2), and pulmonary emphysema (n=1).

Before intervention

Compliance

At the first appointment, 84% (n=16) of the individuals affirmed that their compliance with inhaled therapy was excellent, taking the medication as prescribed every day.

Therapeutic Inhaler Classes

The majority of the patients were using a combination of a long-acting muscarinic antagonist (LAMA) and a long-acting beta-agonist (LABA), represented by 36.8% (n=7). The individuals using LAMA plus LABA and inhaled corticosteroid (ICS) were represented in the same proportion as those using LABA plus ICS, represented by 21.1% each (n=4). Patients using only LAMA therapy were represented by 15.8% (n=3) and LABA by 5.3% (n=1).

Inhalation Technique

Approximately two-thirds of the patients failed at least one step of the inhalation technique. Regarding the steps required for correct inhalation technique (a total of eight steps), at the first visit, 21% (n=4) of the individuals completed six steps, 47% (n=9) completed seven steps, and 32% (n=6) completed all eight steps. The most prevalent error was inadequate apnea time after inhalation of the drug, present in 68% (n=13) of the individuals, followed by an absent forced expiration before inhalation (11%, n=2), shallow inspiration during inhalation (5%, n=1), and failure to confirm the full dose after inhalation (specific step of the Breezhaler device; 5%, n=1).

Reported Symptoms

No individual reported dyspnea at rest. Sixteen percent (n=3) reported dyspnea on mild exertion, 21% (n=4) on moderate exertion, and 42% (n=8) on severe exertion; 21% (n=4) reported no complaints of dyspnea. Regarding other COPD symptoms, 37% (n=7) of the individuals had a daily cough, and 42% (n=8) reported having sputum.

CAT Questionnaire

At the first visit, the CAT questionnaire score ranged from 0 to 21, with a median of 8.

mMRC Questionnaire

At the first visit, the mMRC questionnaire score varied between 1 and 3, with a mean of 1.74.

After intervention

Compliance

In the second appointment, there was a 5% improvement (n=1) in compliance.

Therapeutic Inhaler Classes

All patients maintained the same inhaled therapy between appointments.

Inhalation Technique

Regarding the steps required for correct inhalation technique, 5% (n=1) of the individuals completed six steps, 16% (n=3) completed seven steps, and 79% (n=15) completed eight steps.

The errors still present were inadequate apnea time after inhalation of the drug (11%, n=2), followed by an absent forced expiration before inhalation (11%, n=2), and failure to confirm the full use of the dose after inhalation (specific step of the Breezhaler device; 5%, n=1).

**Figure 2:**
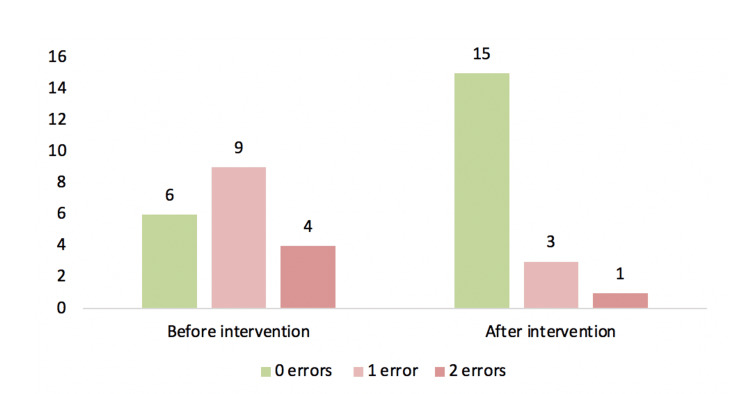
Number of errors made in the inhalation technique before and after intervention.

**Figure 3:**
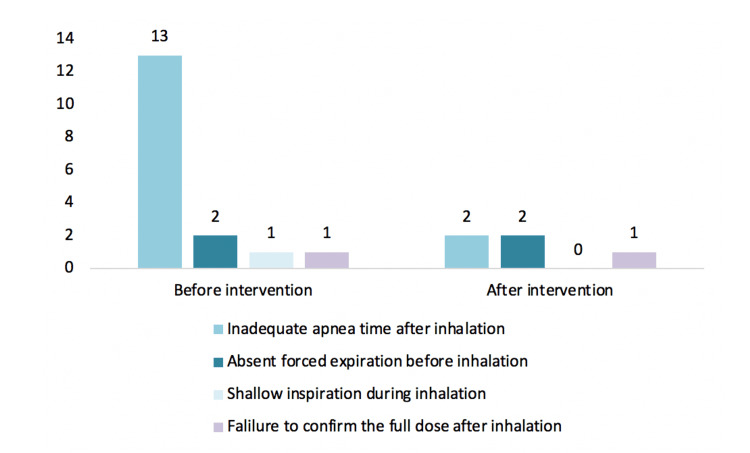
Number and type of failed steps made by the patients during inhalation.

In Figure 2, the investigators compared the errors in inhalation technique before and after the intervention, and in Figure 3, they exposed the failed steps during this technique.

Reported Symptoms

No individual reported dyspnea at rest, 16% (n=3) reported dyspnea on mild exertion, 21% (n=4) on moderate exertion, 21% (n=4) on severe exertion, and 42% (n=8) reported no complaints of dyspnea.

Thirty-two percent (n=6) of the individuals had a daily cough, and 32% (n=6) reported having sputum.

CAT Questionnaire

In the second visit, the CAT questionnaire score ranged between 0 and 21, with a median of 4.

mMRC Questionnaire

In the second visit, the mMRC questionnaire score ranged between 1 and 4, with a mean of 1.63.

Statistic analysis

Using the Wilcoxon signed-rank test, we found statistically significant differences in the number of errors (test statistic = 5.500, p-value = 0.007) and the results from the CAT questionnaire (test statistic = 23.500, p-value = 0.021) before and after the intervention. However, there was no evidence of a significant change in the results of the mMRC questionnaire (test statistic = 2.500, p-value = 0.625) (Table 2).

**Table 2:** Statistical analysis using the Wilcoxon Signed-Rank Test with a significance level set at 5% (p-value < 0.05). CAT: COPD Assessment Test; mMRC: modified Medical Research Council.

	CAT questionnaire	mMRC questionnaire	Number of errors in the inhalation technique
P-value	0.021	0.625	0.007
Statistic	23.500	2.500	5.500

**Figure 4:**
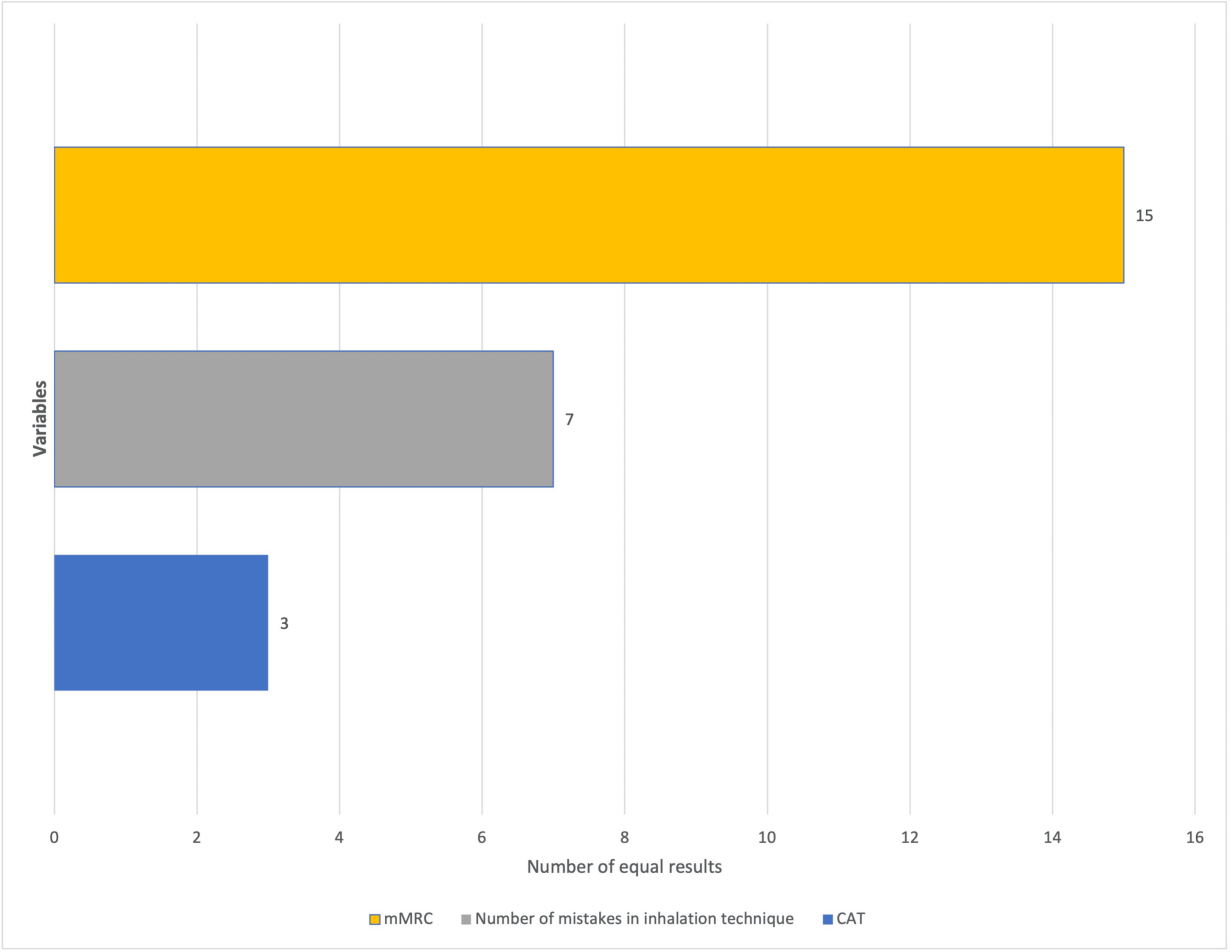
The number of equal results in each variable before and after intervention.

To utilize the aforementioned test, unaffected results before and after the intervention were removed, as depicted in Figure 4.

## Discussion

Inhalation therapy is essential for adequate control of COPD. The main objective of this study was accomplished and surpassed, observing an improvement in inhalation technique in 47% of the patients (>20%).

Regarding the assessment of symptoms through the application of the CAT questionnaire, the median score decreased from 8 to 4. In terms of the mMRC, only one user worsened his or her dyspnea categorization, three improved, and the remainder were classified the same way.

It is important to highlight the difference in sensitivity between the two assessment scales used. The mMRC scale has a sensitivity of 28% [14], while the CAT scale has a significantly higher sensitivity of 78% [15]. This variation may influence the study’s results. Both scales are subjective symptom assessments, but given the higher sensitivity of the CAT, detecting statistical significance with this test is considered more reliable, as it provides greater confidence in evaluating the impact of the intervention.

Despite the limitations of the study, mainly the small sample size that does not allow for statistically significant results, this study observed an improvement in inhalation technique and reported symptoms in patients with COPD at our Health Unit. There is multiple data that proves the need for consistent and correct use of inhalers in people with COPD, and a direct correlation between this correct use and better control of the disease, fewer hospitalizations, and improved quality of life [1,5,6,8]. This could ultimately diminish the economic burden of the disease and the mortality associated with COPD exacerbations.

Strengths and limitations of the study

The use of a random sequence generator helped prevent selection bias, and applying a consistent script across the research team (for phone calls and appointments) along with validated questionnaires minimized performance bias. These methodological strengths, combined with the use of the highly sensitive CAT test, enhance the robustness of our results.

On the other hand, the small sample size can lead to imprecision, since the number of participants was not representative of all COPD patients in this Health Unit. As an intervention project, the study didn't aim to be representative but to intervene and have a positive impact on the correct use of inhalers in people with COPD.

The significant loss to follow-up and withdrawals (41 out of 60 participants) may have introduced attrition bias, which is a recognized limitation of the study.

## Conclusions

Family doctors play a crucial role in the long-term care and management of chronic conditions, consistently monitoring and assessing patients while striving to optimize treatment compliance. This study highlights the impact of targeted appointments focused on managing respiratory diseases, specifically COPD, in improving respiratory symptoms and disease control. Notably, between the first and second visits, patients demonstrated improved inhalation techniques and symptomatic relief, underscoring that regularly reviewing the correct inhalation technique at each visit might be essential for ultimately improving patients' quality of life.

Moreover, this study raises an important question about the potential need for specialized appointments within primary care settings. While family doctors are essential for managing chronic conditions, the complexity of diseases like COPD may necessitate a more focused approach to ensure optimal care. The authors also consider it important to routinely implement the determination of oxygen saturation at all appointments. Specialized appointments could allow for more in-depth monitoring, tailored interventions, and targeted education, which might improve disease management and patient outcomes.

## Appendices

Appendix 1

**Table 3:** Modified Medical Research Council (mMRC) dyspnea scale.

Grade	Description of breathlessness
0	I only get breathless with strenuous exercise
1	I get short of breath when hurrying on level ground or walking up a slight hill
2	On level ground, I walk slower than people of the same age because of breathlessness, or have to stop for breath when walking at my own pace
3	I stop for breath after walking about 100 yards (91 meters) or after a few minutes on level ground
4	I am too breathless to leave the house or I am breathless when dressing

**Table 4:** COPD Assessment Test (CAT).

COPD Assessment Test (CAT)															
Your name: ___________															
Today’s date: __________															
This questionnaire will help you and your healthcare professional to measure the impact that COPD (Chronic Obstructive Pulmonary Disease) is having on your wellbeing and daily life. Your answers and test score can be used by you and your healthcare professional to help improve the management of your COPD and gain the greatest benefit from the treatment. For each item below, place a mark (X) in the best that best describes your current situation. Please ensure that you only select one response for each question.															
Example:															
I am very happy.	0		1		2		3		4		5		I am very sad.		
Answer														Score	
I never cough.	0	1		2		3		4		5		I cough all the time.			
I have no phlegm (mucus) on my chest at all.	0	1		2		3		4		5		My chest is full of phlegm (mucus).			
My chest does not feel tight at all.	0	1		2		3		4		5		My chest feels very tight.			
When I walk up a hill or a flight of stairs I am not out of breath.	0	1		2		3		4		5		When I walk up a hill or a flight of stairs I am completely out of breath.			
I am not limited to doing any activities at home.	0	1		2		3		4		5		I am completely limited to doing activities at home.			
I am confident leaving my home despite my lung condition.	0	1		2		3		4		5		I am not confident leaving my home at all because of my lung condition.			
I sleep soundly.	0	1		2		3		4		5		I do not sleep soundly because of my lung condition.			
I have lots of energy.	0	1		2		3		4		5		I have no energy at all.			
Total score:															
A COPD assessment test was developed by an interdisciplinary group of international COPD experts with support from GSK. GSK's activities in connection with the COPD assessment test are monitored by a supervisory council that includes external, independent experts, one of which is chair of the council. CAT, the COPD assessment test and the CAT logo are trademarks that belong to the GSK group of companies. ©2009 GSK. All rights reserved.															

## References

[1] (2024). Golden Initiative for Chronic Obstructive Lung Disease. Golden Strategy for the Diagnosis, Management, and Prevention of Chronic Obstructive Pulmonary Disease (2023 Report). https://goldcopd.org.

[2] (2024). Direção-Geral da Saúde. NOC 005/2019 - Diagnóstico e Tratamento Da Doença Pulmonar Obstrutiva Crónica No Adulto. https://www.dgs.pt/.

[3] Ribeiro S, Cardoso CS, Valério M, Machado J, Costa J, Rodrigues C, Rebelo-Marques A (2022). Confirmatory evaluation of the modified Medical Research Council questionnaire for assessment of dyspnea in patients with chronic obstructive pulmonary disease in Portugal. Acta Med Port.

[4] Pimenta Valério M, Ribeiro S, Seiça Cardoso C, Machado J, Costa J, Rodrigues C, Rebelo-Marques A (2022). European Portuguese language and cultural validation of the chronic obstructive pulmonary disease assessment test. Acta Med Port.

[5] Bárbara C, Rodrigues F, Dias H (2013). Chronic obstructive pulmonary disease prevalence in Lisbon, Portugal: the burden of obstructive lung disease study. Rev Port Pneumol.

[6] Kaplan A, Rodriguez MR, Williams S (2023). Achieving earlier diagnosis of COPD. Desktop Helpers.

[7] Pham HQ, Pham KH, Ha GH, Pham TT, Nguyen HT, Nguyen TH, Oh JK (2024). Economic burden of chronic obstructive pulmonary disease: a systematic review. Tuberc Respir Dis (Seoul).

[8] Chakrabarti S (2014). What's in a name? Compliance, adherence and concordance in chronic psychiatric disorders. World J Psychiatry.

[9] Lareau SC, Yawn BP (2010). Improving adherence with inhaler therapy in COPD. Int J Chron Obstruct Pulmon Dis.

[10] Barbosa MJ, Castro D, Costa R (2023). Guia Prático de Gestão da DPOC nos Cuidados de Saúde Primários. https://gresp.pt/ficheiros/recursos/guias-praticos/guia-pratico-de-gestao-da-dpoc-2023.pdf.

[11] Jones PW, Brusselle G, Dal Negro RW (2012). Patient-centred assessment of COPD in primary care: experience from a cross-sectional study of health-related quality of life in Europe. Prim Care Respir J.

[12] (2024). Magalhães DV (2020). Proporção de doentes com DPOC em Portugal sob a perspetiva da
Medicina Geral e Familiar. https://estudogeral.uc.pt/handle/10316/97752.

[13] Sociedade Portuguesa de Alergologia e Imunologia (2024). Sociedade Portuguesa de Alergologia e Imunologia Clínica "Checklist inaladores". https://www.spaic.pt.

[14] Gustafsson D, Elmberg V, Schiöler L, Jensen D, Ekström M (2023). The modified Medical Research Council scale misclassifies exertional breathlessness among people referred for exercise testing. ERJ Open Res.

[15] Al Wachami N, Boumendil K, Arraji M (2024). Evaluating the effectiveness of the COPD Assessment Test (CAT) in screening for chronic obstructive pulmonary disease. Int J Chron Obstruct Pulmon Dis.

